# Sustained Viral Response and Hematological Adverse Events During Chronic Hepatitis C Infection Treatment

**DOI:** 10.5812/hepatmon.831

**Published:** 2012-02-29

**Authors:** Mortada El-Shabrawi, Mona Isa

**Affiliations:** 1 Department of Pediatrics, Faculty of Medicine, Cairo University, Cairo, Egypt

**Keywords:** Infection, Hepatitis C, Treatment

Dear Editor,

Hepatitis C virus (HCV), as a causative agent of chronic liver disease, has infected approximately 175 million people (almost 3%) of the world's population; and 3 to 4 million new cases are added to this figure annually [1]. Chronic HCV infection may progress to severe outcomes in the form of cirrhosis and hepatocellular carcinoma (HCC) [2]. Currently, there is no effective HCV vaccine on the horizon due to a lack of a susceptible small animal model, an absence of neutralizing antibodies, and a high degree of viral genomic diversity and mutagenicity; therefore, successful treatment of HCV infection is very much needed. A few years ago, the standard of care (SOC) for chronic HCV infection consisted of subcutaneous injection of conventional Interferon (IFN)-α-2, 3 times per week, plus an oral, daily dose of Ribavirin (RBV) for 24 to 48 weeks [2][3]. This therapy is not ideal because of a very low sustained virologic response [(SVR) i.e., HCV RNA undetectable 6 months after the end of treatment]. The current SOC consists of pegylated INF-α-2 once a week plus daily RBV for 24 to 72 weeks [4]. This treatment aims to achieve a high SVR [5]; however, different people respond differently to this SOC regimen depending on many factors, particularly the age, sex, and ethnicity of the patient; the duration of infection; adiposity; the degree of aminotransferase elevation; HCV genotype; pretreatment viral load; and single nucleotide polymorphisms of interleukin-28B gene [6]. Recently, oral protease inhibitors ( e.g., telaprevir or boceprevir) have been added as direct-acting antivirals to the SOC treatment as a triple therapy, particularly in patients with HCV genotype 1 [7]. We were interested in Pawlowska et al.'s study, which examined correlations between the hematological adverse events and the SVR in children undergoing therapy for chronic HCV infection [8]. Specifically, Pawlowska et al. assessed the interdependence of the SVR and the hematological characteristics (leukocyte count, platelet count, and hemoglobin levels) in patients with chronic HCV infection during treatment with IFN and RBV. They divided their sample of children into two groups: patients in Group I were treated with conventional IFN-α-2b plus RBV, and patients in Group II were treated with pegylated IFN-α-2b plus RBV. They concluded that mild decreases in hemoglobin levels, leukocyte counts, and platelet counts during treatment with IFN and RBV in children with chronic HCV infection may be factors associated with SVR induction. Hemoglobin levels decreased significantly in patients who achieved SVR compared to the nonresponders in both groups. In a similar study by Sievert et al. [9] the virologic responses were also higher in anemic patients than in patients who did not develop anemia. After 12 weeks of therapy, the leukocyte and platelet counts were significantly lower in children treated with pegylated IFN-α-2b plus RBV than in those treated with conventional IFN plus RBV [8]. The hematological toxicity that occurs during therapy can result in modifications in dosage or even, in the worst-case scenario, withdrawing INF therapy, which decreases the chances of successful therapy and increases the risk of impaired liver function with cirrhosis and HCC as potential consequences [10]. Two studies have suggested that pegylated INF therapy combined with Danazol could be used to effectively treat patients suffering from HCV-related thrombocytopenia; this combined therapy avoids temporarily reducing or definitively stopping pegylated INF treatment and increases platelet levels [10][11]. The literature has clearly established that the rate of SVR with pegylated INF and RBV is comparatively higher in patients with genotypes 2 and 3 (80%) than in patients with genotypes 1 or 4 (40-50%) [4] . Despite achieving a higher SVR rate, one of the drawbacks of pegylated INF is that it is least 25 times more expensive than conventional interferon, making it unaffordable for many poor people in developing countries [5]. Suwantarat et al. found that chronic HCV-infected patients with SVR had significantly lower white blood cell and platelet counts at the end of treatment compared to those without SVR. These findings suggest that patients who develop leucopenia or thrombocytopenia during interferon treatment respond well to the therapy, and these side effects, if not severe, may not be reasons to withhold or reduce the dose of the treatment. They hypothesized that the greater cytopenia might be an indication of greater tumor necrosis factor activity in a specific treatment recipient, which translates into a higher SVR [12].
